# Inflammatory response to strenuous muscular exercise in man

**DOI:** 10.1155/S0962935193000468

**Published:** 1993

**Authors:** G. Camus, G. Deby-Dupont, C. Deby, A. Juchmès-Ferir, J. Pincemail, M. Lamy

**Affiliations:** 1 Research Associate, FNRS Laboratory of Human Applied Physiology, ISEPK, B21 University of Liège Sart Tilman Liège 4000 Belgium; 2 Center for the Biochemistry of Oxygen Institute of Chemistry, B6 University of Liège Sart Tilman Liège 4000 Belgium; 3 Department of Clinical Biology University of Liège Sart Tilman Liège 4000 Belgium; 4 Department of Anesthesiology CHU, B35 University of Liège Sart Tilman Liège 4000 Belgium

## Abstract

Based on the humoral and cellular changes occurring during strenuous muscular work in humans, the concept of inflammatory response to exercise (IRE) is developed. The main indices of IRE consist of signs of an acute phase response, leucocytosis and leucocyte activation, release of inflammatory mediators, tissue damage and cellular infiltrates, production of free radicals, activation of complement, and coagulation and fibrinolytic pathways. Depending on exercise intensity and duration, it seems likely that muscle and/or associated connective tissue damage, contact system activation due to shear stress on endothelium and endotoxaemia could be the triggering mechanisms of IRE. Although this phenomenon can be considered in most cases as a physiological process associated with tissue repair, exaggerated IRE could have physiopathological consequences. On the other hand, the influence of several factors such as age, sex, training, hormonal status, nutrition, anti-inflammatory drugs, and the extent to which IRE could be a potential risk for subjects undergoing intense physical training require further study.


Mediators of Inflammation 2, 335–342 (1993)

### Inflammatory response to strenuous muscular exercise in man

G. Camus,[1,2,CA] G. Deby-Dupont,[4] C. Deby,[2] A. Juchmès-Ferir,[3] J. Pincemail[2] and M. Lamy[2,4]

[1]Research Associate, FNRS, Laboratory of Human Applied Physiology, ISEPK, B21, [2]Center for the Biochemistry of Oxygen, Institute of Chemistry, B6, [3]Department of Clinical Biology, [4]Department of Anesthesiology, CHU, B35, University of Liège, Sart Tilman, 4000 Liège, Belgium

[CA] Corresponding Author

**BASED on the humoral and cellular changes occurring during strenuous muscular work in humans, the concept of inflammatory response to exercise (IRE) is developed. The main indices of IRE consist of signs of an acute phase response, leucocytosis and leucocyte activation, release of inflammatory mediators, tissue damage and cellular infiltrates, production of free radicals, activation of complement, and coagulation and fibrinolytic pathways. Depending on exercise intensity and duration, it seems likely that muscle and/or associated connective tissue damage, contact system activation due to shear stress on endothelium and endotoxaemia could be the triggering mechanisms of IRE. Although this phenomenon can be considered in most cases as a physiological process associated with tissue repair, exaggerated IRE could have physiopathological consequences. On the other hand, the influence of several factors such as age, sex, training, hormonal status, nutrition, anti-inflammatory drugs, and the extent to which IRE could be a potential risk for subjects undergoing intense physical training require further study.**

**Key words:** Acute phase response, Exercise, Free radicals, Inflammatory mediators, Leucocyte, Muscle damage

### Introduction

The concept of exercise induced inflammatory reaction in man has evolved from studies conducted in the 1970s, showing that strenuous exercise was followed by significant increases in plasma levels of several glycoproteins which are typical markers of the acute phase response to inflammatory or infectious diseases.[1–3] These findings were confirmed by more recent experimental work where changes in plasma levels of acute phase reactants[4–8] and trace minerals[5,9] have been reported in subjects submitted to various exercise protocols. By analogy with pathological disorders,[10,11] it was inferred that strenuous exercise could trigger an acute phase response displaying a pattern similar to that found after trauma or during infection.[3–9,12]

Owing to the development and improvement of techniques for studying the humoral and cellular changes occurring in inflammatory states, convincing evidence that this acute-phase response was due to the production of cytokines during exercise was presented.[7,8] The concept of the inflammatory response to exercise (IRE) was further substantiated by subsequent studies based on the measurement of various humoral[6–32] and cellular[33–36] markers of acute inflammation in exercising subjects. Although this concept of IRE is acknowledged at the present time, its underlying mechanisms are poorly understood. Furthermore, its physiological meaning and possible consequences remain open to conjecture. In this review, the authors attempt to summarize current knowledge of the main features of IRE. Some hypotheses on the underlying mechanisms, physiological meaning and consequences of this phenomenon are also presented.

### Main features of the inflammatory response to exercise

Evidence for the occurrence of an inflammatory response to strenuous exercise is obtained from increasing amounts of experimental data showing that the patterns of metabolic and immunologic change induced by exercise resemble those typically observed in inflammatory or infectious diseases. Indices of IRE include signs of an acute-phase response,[1–9,12] leucocytosis[30,37,38] and leucocyte activation,[23,27–33] release of inflammatory mediators,[13–26] tissue damage and cellular infiltrates,[33–36] production of free radicals,[29,39–47] activation of complement,[19,22–24] and coagulation and fibrinolytic cascades.[22,48–51]

*Acute-phase response:* The acute-phase response refers to a constellation of biochemical and endocrine changes induced within the body by various stressors such as infections, traumas, inflammatory processes and some malignant diseases.[11] These stimuli evoke a characteristic pattern of altered hepatocyte protein synthesis resulting in increased

© 1993 Rapid Communications of Oxford Ltd plasma levels of glycoproteins and globulins such as C-reactive proteins, α-1-antitrypsin, fibrinogen, ceruloplasmin, ferritin and haptoglobin, while the synthesis of albumin is decreased.[9,10] Plasma zinc and iron concentrations decrease whereas plasma copper concentration rises.[5,9] The acute-phase response is also characterized by a neutrophilic leucocytosis resulting from granulocyte demargination and release from the bone marrow into the blood.[10] Most of these metabolic changes were also observed in subjects submitted to strenuous exercise. Increased plasma levels of globulins, glycoproteins, acute-phase proteins, transferrin, creatine kinase, fibrinogen and ferritin were reported.[1–3,8,9] Acute and chronic hypoferraemia and hypozincaemia were found after sustained strenuous exercise or long-term training.[9] These humoral changes are thought to be mediated by increased cytokine secretion occurring during exercise.[7–9]

*Leucocyte mobilization and activation:* The effects of exercise on peripheral white blood cell count (WBC) has been the subject of numerous studies based on various experimental protocols.[30,37,38] Most of these studies have shown that muscular work induces an immediate leucocytosis mainly due to demargination of cells from the non-circulating leucocyte pool.[38] Increased secretion of catecholamines and cortisol, and haemodynamic changes seem to play the major role in this exercise induced leucocytosis.[38] The magnitude and pattern of WBC change over time depend, among other factors, on exercise intensity and duration.[38] These parameters also influence the differential counts.[38] In agreement with data from the literature, the authors found that the increase in WBC during short-term dynamic exercise results from elevated lymphocyte and neutrophil counts.[37] While the number of circulating lymphocytes tended to decrease to resting levels within the first 20 min after exercise, PMN counts remained practically unchanged after 20 min recovery.[23,37] During exercise of longer duration, such as the marathon, the leucocytosis primarily results from elevated polymorphonuclear cell count.[38] The marked and persistent exercise induced rise in PMN count, mimicking that observed in the acute-phase response to pathological disorders, led several authors to consider this neutrophilia as a potential sign of inflammation.[36] Nevertheless, the extent to which the leucocytosis of exercise could modulate IRE remained to be established. Sprenger *et al.*[26] hypothesized that elevated cytokine synthesis during exercise could be due, at least in part, to increased cellular contacts between leucocytes and other cells. With this in view, enhanced leucocyte count could be involved in cytokine production during muscular work.

As outlined above, stress hormones and haemodynamic changes appear to be the principal factors responsible for the exercise induced changes in WBC. Whether inflammatory mediators and chemotactic compounds released from injured tissue play a role in the immediate and delayed exercise induced leucocytosis is unknown. These attractive hypotheses warrant further study.

Leucocyte activation is another typical feature of IRE. Once activated, these cells release specific compounds in their environment. Based on the measurement of these compounds as specific markers of white blood cells triggering, a number of studies provided evidence for exercise induced activation of several cell lines.[23,27–32] As stated above, significant increases in plasma and urinary concentration of cytokines were reported after muscular work, demonstrating monocyte/macrophage activation.[20,26] Neutrophil triggering was evidenced by enhanced plasma levels of polymorphonuclear elastase,[30,32] elastase-α-1-proteinase complex[20,27] and myeloperoxidase.[23,29,31,32] Increased plasma concentrations of soluble interleukin 2 (IL-2) receptor, neopterin[20] and plasma IFN activity[25] has been reported in exercising subjects, showing that T-lymphocytes and macrophages were also activated. Histological examination of muscle fragments provides further evidence for infiltration of damaged tissue by activated leucocytes.[33–35] Despite this rather large body of data, the mechanisms underlying the exercise induced leucocyte activation and its consequences are poorly understood. Based on the results of a recent study, the authors hypothesized that C5a anaphylatoxin —an activated complement component—could be involved in this phenomenon.[23]

*Complement activation:* Owing to inappropriate methods of investigation, several studies have failed to demonstrate complement activation during exercise.[24] More recent researches based on the measurement of anaphylatoxins by means of sensitive radioimmune assays provided evidence for complement activation during strenuous exercise. Increased plasma levels of C3a, C4a and C5a anaphylatoxins have been reported in human subjects submitted to strenuous exercise.[19,22–24] However, the underlying mechanisms of exercise induced complement activation and its functional significance remain hypothetical.

From the significant rises in C4a, the correlation of changes in C3a with changes in C4a and C4 haemolytic activity, Smith *et al.*[24] suggested that the classical pathway of complement was activated by immune complexes or by nonspecific factors such as C-reactive proteins, trypsin or plasmin. As pointed out by these authors, C3a could also be generated *via* activation of the alternative pathway or by the direct action of cathepsin G, trypsin and plasmin on C3. Based on the data of the literature, it seems that these mechanisms are feasible. Nevertheless, a recent study by Thomsen *et al.*[52] showed that levels of complement receptor type one, circulating immune complexes and complement C3 split products C3d and C3c are not changed by short-term physical exercise or training. Although these data are not incompatible with the occurrence of anaphylatoxin production during exercise, the lack of change in circulating immune complexes suggests that exercise induced complement activation probably relies on other mechanisms.

Interestingly, the authors found that constant-load cycling exercise (20 min at 80% $VO_2max$) induced significant and parallel increases in plasma concentration of MPO and C5a anaphylatoxin.[23] These variables returned to baseline levels after 20 min recovery. The similar time course of MPO and C5a changes, and the significant relationship between these two variables argue for the involvement of C5a anaphylatoxin in the process of PMN degranulation during exercise. Preliminary findings presented by Smith *et al.*[24] on exaggerated C3a production in three asthmatic runners suggest that anaphylatoxins could be involved in the exercise induced asthma. However, given the small number of subjects, these findings are at most suggestive.

In conclusion, it thus appears that factors involved in complement activation and the physiological consequences of this phenomenon remain to be elucidated.

*Cytokine production:* The occurrence of exercise induced cytokine production was first suggested by Cannon and Kluger.[4] These authors showed that human plasma taken after 1 h exercise at 60% $VO_2max$ exhibited endogenous pyrogen activity when injected into rats. From this observation, they concluded that exercise was accompanied by interleukin-1 (IL-1) release. Their conclusion was confirmed later in subsequent studies based on more direct methods of IL-1 assay. Using a specific immunochemical method, Cannon *et al.*[12] detected a significant increase in IL-1$\beta$ in muscle tissue samples taken from subjects previously submitted to a 45 min downhill run at 75% $VO_2max$. Since that time, an increasing number of experimental researches clearly demonstrated that strenuous exercise induces not only the release of IL-1, but also the production of other cytokines such as IL-6,[10,26] tumour necrosis factor (TNF)[20,26] and interferon (IFN).[25,26] Although the role played by these pro-inflammatory molecules in the host defence system and in the pathogenesis of trauma and in inflammatory diseases is at present well established, the physiological meaning of these humoral changes in healthy exercising subjects is still obscure. These compounds—especially IL-1, IL-6 and TNF—could act in concert to initiate an acute-phase response involved in the process of repair of damaged tissues by exercise. Consistent with this view is the appearance of acute-phase reactants in blood following strenuous muscular work (see above) and the significant relationship found by Cannon *et al.*[7] between urinary 3-methylhistidine excretion—taken as an indication of muscle proteolysis—and *in vitro* secretion of IL-1$\beta$ and prostaglandin $E_2$ ($PGE_2$) from mononuclear cells taken from sedentary subjects after vigorous exercise. These findings support the concept that these mononuclear cell products were involved in muscle proteolysis. These authors proposed a hypothetical sequence of events where tissue fragments from damaged muscles could activate the complement system, which in turn would prime monocytes for activation by these tissue fragments. As a result, these cells secrete increased amounts of IL-1$\beta$ and $PGE_2$ which regulate muscle proteolysis. Based on our current knowledge of leucocyte functions in pathological situations, this attractive hypothesis appears tenable. However, a recent study by Smith *et al.*[53] showed that moderate endurance exercise could trigger an acute-phase response in untrained subjects without significant changes in the blood level of cytokine and creatine kinase (CK), taken as an indirect index of muscle damage. Sprenger *et al.*[26] examined cytokine production in well-trained runners covering a distance of 20 km and found that, except for IL-6, cytokines were not detected in plasma but were present in urine. Moreover, they reported marked increases in urinary concentration of IFN-$\gamma$, TNF-$\alpha$, IL-6 and IL-1$\beta$ after exercise. From the small increase in plasma CK activity and the lack of correlation between this variable and TNF$\alpha$ in urine, they suggested that muscle damage was not the underlying mechanism of the exercise induced cytokine secretion observed in their subjects. From these findings, it is thus likely that other factors are involved in the exercise induced cytokine production and related acute-phase response.

Two separate studies have shown that long-term strenuous exercise, such as a triathlon competition[54] and a 89.4 km run,[55] were accompanied by significant increases in the blood level of lipopolysaccharide (LPS), so that it can be hypothesized that endotoxaemia could trigger transient immunological and inflammatory reactions following a pattern similar to that found at the early stage of sepsis.[31] However, the occurrence of such an endotoxaemia has not been demonstrated in exercise of shorter duration, and appears unlikely, especially when exercise is achieved by well-trained subjects in normal environmental conditions. In agreement with this view is the lack of endotoxaemia in trained runners who took part in a 21.1 km race 3 weeks after a 89.4 km road race.[55] It should be kept in mind, however, that most of the runners with elevated plasma levels of endotoxin had decreased anti-endotoxin IgG concentrations.[55]

The underlying mechanisms of exercise induced endotoxaemia, its relation to physical fitness, antibody production, and performance and environmental conditions should be investigated further.

*Production of arachidonic acid derived compounds:* The effects of muscular work on arachidonic acid (AA) metabolism is well documented. This component of plasma membrane phospholipids is liberated from cell membranes by the ubiquitous lipolytic enzyme phospholipase $A_2$ ($PLA_2$). Once liberated into the cell, AA can be converted to various endoperoxides by the cyclooxygenase and lipoxygenase enzymes. AA and various prostaglandins (PG) derived from the cyclooxygenase pathway of AA metabolism have been found to accumulate in skeletal muscle during contraction.[16,17] Increased blood levels of PG and 6-keto $PGF_{1a}$, the stable metabolite of $PGI_2$ (prostacyclin), have been reported in exercising subjects.[14,15] From experiments showing that repetitive contractions were accompanied by enhanced PG concentration in venous effluent from skeletal muscle,[13] it thus appears that these eicosanoids are released from working muscles into the blood. The mechanisms responsible for $PLA_2$ activation and subsequent conversion of AA into PG during exercise are unclear. An imbalance between muscle oxygen supply and demand has been shown to be an important stimulus for exercise induced PG production.[16]

According to Symons *et al.*[16] blood vessel walls are the main sites for PG production in cat gastrocnemius muscle after 30 s of high-intensity static contraction. Based on a previous study where bradykinin, a stimulator of $PLA_2$, was found to be released in the venous effluent from the cat hindlimb,[18] Symons *et al.*[16] suggested that this compound could be involved in the exercise induced eicosanoid production in skeletal muscle. Although this hypothesis seems tenable, the possibility that other inflammatory mediators such as cytokines and complement anaphylatoxins could play a role in increased PG synthesis through leucocyte activation cannot be ruled out.

Previous data from the literature suggest that PG may well contribute to regional vasodilatation in muscle during dynamic contractions and sensitize groups III and IV afferent nerve endings to contraction induced bradykinin response.[16] Sensitization of these afferent nerve endings appears to be required for the full reflex response of the cardiovascular system to static contraction.[16] Primed monocytes/macrophages are another potential source of PG during exercise. As outlined above, these cells were found to be activated by exercise. Upon activation by inflammatory stimuli at the site of muscle injury, invading macrophages can secrete large amounts of PG in their surroundings.[36] While the involvement of these cells in delayed PG synthesis and associated delayed onset muscle soreness (DOMS) appears plausible, their role in the early PG production remains to be demonstrated.

In contrast, a recent study by Hasson *et al.*[56] gives support to the hypothesis that products of cyclooxygenase activity play a role in the painful manifestations associated with DOMS. Hasson *et al.*[56] studied the effects of a powerful anti-inflammatory drug (ibuprofen) that inhibits the enzyme cyclooxygenase on muscle damage, soreness and decreased performance induced by a strenuous eccentric exercise bout. They showed that ibuprofen ingestion 4 h before exercise significantly decreased muscle soreness perception, and the decline in isometric, concentric and eccentric torque following an eccentric exercise test. Interestingly, muscle damage assessed by plasma CK measurements was unaffected by the drug. The findings of Hasson *et al.*[56] prompted us to verify whether a steroidal anti-inflammatory agent could reduce DOMS symptoms and decrease the transient exercise induced release of elastase and myeloperoxidase. This type of compound, such as methylprednisolone, is thought to exert its anti-inflammatory effects by inducing the synthesis of lipocortins which, in turn, inhibits phospholipase $A_2$ production of arachidonic acid. Therefore, we studied the effects of a single oral dose of methylprednisolone on the exercise induced changes in plasma levels of elastase and myeloperoxidase in four healthy male subjects submitted to a 20 min downhill run at 60% $VO_{2max}$ on an inclined treadmill (−20% grade).[57] Exercise started 3 h after oral absorption of a placebo or a single dose of 32 mg methylprednisolone. While the exercise induced increase in plasma MPO concentration was the same in both trials, the rise of plasma elastase level accompanying exercise was markedly amplified by methylprednisolone. On the other hand, there were no effects of methylprednisolone on subjective DOMS symptoms. These preliminary results showed that a single dose of a steroidal anti-inflammatory agent taken 3 h before eccentric exercise did not decrease the release of two specific markers of PMN activation. Given the small number of subjects who took part in this study, the lack of effect of methylprednisolone on DOMS

remains at most suggestive and should be verified by further experiments. Nevertheless, from these findings and other data from the literature,[58–60] it thus appears that the effects of anti-inflammatory drugs on exercise induced muscle damage and related consequences are a matter of controversy. Depending on exercise protocols, nature and amount of drug used, animal species and conditions of drug administration (prophylactically, before exercise; therapeutically, after exercise)[56] the effects of agents that inhibit AA metabolism on several aspects of IRE could differ widely. The possible interference of these drugs with tissue repair after exercise is unclear and obviously needs further study.

*Free radical production:* Since the original work by Dillard *et al.*,[40] an increasing body of data showed that strenuous exercise increases the production of free radicals.[39–47] Using the measurement of various products of lipid peroxidation such as pentane in expired air samples, and conjugated dienes and malondialdehyde in blood or muscle tissue samples, several authors provided evidence for the occurrence of increased lipid peroxidation, and therefore, enhanced production of free radicals during muscular work.[42] Other indirect evidence of this phenomenon stemmed from the decrease of tissue and blood antioxidant content reported in several studies. Using an electron paramagnetic resonance method, Jackson *et al.*[41] first demonstrated the enhanced production of free radicals in muscle stimulated to contract. Despite this convergent body of experimental results, the exact mechanisms of radical production during exercise are poorly understood. Increased turnover of semiquinone in mitochondria,[42] xanthine oxidase catalysed reactions,[42,61,62] haemoglobin auto-oxidation,[63] phagocyte and PMN activation,[64–66] and eicosanoid production from AA by the cyclooxygenase and lipoxygenase enzymes[67] are potential sources of free radicals during exercise. To date, the relative importance of these mechanisms and their precise involvement in exercise induced lipid peroxidation are unknown.

In connection with IRE, several studies were conducted to verify whether free radicals or oxidant species from activated leucocytes or other sources could damage muscle structures and lead to the release of muscle proteins in blood. Maughan *et al.*[45] showed that a 45 min downhill run resulted in significant and sustained increases in lipid peroxide concentration and muscle enzyme release in blood. Because the greatest increases in lipid peroxide concentration were found in subjects with the highest enzyme release, they suggested that activated PMN, macrophages and monocytes by muscle damage could be responsible for the delayed production of free radicals. Sumida *et al.*[46] reported that tocopherol supplementation reduced the blood level of malondialdehyde and the leakage of muscle enzymes after an incremental exercise to exhaustion. Cannon *et al.*[6] found a significant relationship between plasma CK concentration and the spontaneous production of superoxide anion by isolated PMN *in vitro* after strenuous eccentric exercise. These findings led to the assumption that free radicals produced by activated leucocytes could be involved in exercise induced muscle damage. However, subsequent studies based on antioxidant supplementation and vitamin E deficiency yielded puzzling results. Warren *et al.*[68] compared various indices of muscle damage induced by walking downhill in two groups of rats receiving either a normal or a vitamin E supplemented diet. While vitamin E supplementation decreased susceptibility of muscles to oxidant stress, reduction in maximal tetanic force, number of intact fibres per square millimeter, elevations in muscle glucose 6-phosphate dehydrogenase and plasma creatine kinase activities did not differ significantly between the two groups of animals.

Amelink *et al.*[69] studied the effects of a vitamin E deficient diet on histological and biochemical indices of muscle damage in rats submitted to a treadmill exercise. They found that increases in plasma CK activity and alteration of the cyto-architecture of the muscle fibres were markedly enhanced in the vitamin E deficient rats. Interestingly, they showed a sex difference in the susceptibility to exercise induced muscle damage of vitamin E deficient rats. Male rats exhibited a larger CK response and more marked histopathological changes than females. Based on the results of previous work, they attributed this sex difference to a protective effect of oestradiol in female rats. From the results of an *in vitro* study by Frei *et al.*,[70] showing that antioxidants of human plasma samples were consumed in a temporal sequence in the presence of activated PMN, it has been inferred that toxic oxygen species and chlorinated compounds released from circulating PMN could alter the blood antioxidant status and might be involved in lipid peroxidation phenomena during exercise. These authors noticed that hydroperoxides derived from plasma phospholipids only appeared after ascorbate was completely consumed. The oxidation of ascorbate occurred despite the presence of other antioxidants in high concentrations in plasma.[70] The increase of erythrocyte susceptibility to *in vitro* peroxidation[43] and the significant decrease in the reduced glutathione content of blood[44] argue for the release of oxidant compounds in blood during exercise. To verify whether transient activation of circulating PMN could lead to oxidation of blood glutathione and plasma ascorbic acid, we studied the effects of concentric (uphill walk) and eccentric (downhill run) exercises of 20 min duration at 60% $VO_2$max on the blood levels of reduced/oxidized glutathione and plasma concentration of ascorbic acid in male subjects.[71] This protocol was based on the results of a recent study where it was found that while uphill walk did not elicit any significant changes in plasma concentrations of polymorphonuclear MPO and elastase following UW, downhill run caused significant increases in the plasma level of these two specific markers of PMN activation.[32] It was found that uphill walk did not cause any significant changes in either plasma MPO or ascorbic acid concentrations. On the contrary, plasma concentration of MPO increased and plasma ascorbic acid decreased significantly during downhill running tests. From the negative relationship between these two variables, we suggested that transient exercise induced PMN activation could be involved, at least in part, in the decrease in plasma ascorbic acid levels accompanying eccentric exercise. The stable blood concentration of reduced and oxidized glutathione during both exercise tests led to the conclusion that the oxidant stress associated with exercise was insufficient to alter the blood levels of these compounds. The unchanged susceptibility of erythrocytes to lipid peroxidation induced *in vitro* by 2,2′ azobis-(2-amidinopropane) dihydrochloride after a 10 km road race despite the occurrence of significant *in vivo* PMN activation during this event further illustrates the efficacy of antioxidant protection of human erythrocytes to the oxidant stress of exercise (unpublished results). Despite the uncertainties concerning the transposition of this finding to the conditions prevailing *in vivo*, it seems reasonable to assume that more strenuous exercise would be required to significantly alter the antioxidant status of blood and lead to detectable amounts of blood derived products of oxidant species attack.

*Cellular infiltrates:* Histological examination of damaged muscle after strenuous exercise clearly demonstrated the appearance of various cells of the immune system in the biopsy samples and provided further evidence for exercise induced leucocyte activation.[33–35] Round *et al.*[35] reported that the predominant cell type was the macrophage, but T-lymphocytes, mainly of the CD4 positive helper/inducer subset, a few B-lymphocytes,[34] and activated PMN[33] were also found. The presence of these cellular infiltrates raised the question of whether the invading cells could be the cause of further tissue injury or the consequence of the damage, scavenging and removing the cellular debris of injured cells. Elegant work by Jones *et al.*[34] shed light on this point. These authors studied the effects of eccentric contractions on muscle CK release, uptake of technetium pyrophosphate—taken as indices of muscle damage—and mononuclear cell infiltration in muscle biopsy samples up to 20 days after exercise. Because tissue infiltration was a maximum well after the time of the peak of enzyme release and of technetium pyrophosphate uptake, they concluded that the invading cells cannot be held responsible for muscle damage. These findings lead to the assumption that the mononuclear cell infiltrates are involved in muscle tissue repair after damaging exercise rather than in amplification of tissue injury. It should be kept in mind, however, that previous studies led support to the concept that products of activated leucocytes, including $PGE_2$, IL-1$\beta$, oxidant species and proteases could contribute to tissue catabolism. Therefore, the possibility that exaggerated leucocyte activation could play a significant role in amplifying muscle damage cannot be ruled out.

*Increase in blood coagulability, fibrinolytic and platelet activity:* Hypercoagulability, enhancement of fibrinolytic potential and platelet activation are other features common to strenuous muscular work and some inflammatory states. The underlying mechanisms of these haemostatic changes are poorly understood. The marked and persistent increase in the von Willebrand factor observed in exercising subjects[50] argued for the occurrence of shear stress damage to endothelial cells. Such damage could increase the coagulability of blood through activation of the contact system by subendothelial collagen exposure.[50] Release of thrombolastins from activated monocytes or damaged tissues, and proteolytic attack of blood components of the coagulation cascade by proteases from activated leucocytes could also participate in the enhancement of blood coagulability.[49–51]

The platelet activation accompanying exercise is reflected by the release of platelet factor 4, b-thromboglobulin in blood, and by the decrease in the threshold of ATP necessary to induce aggregation.[49] Among the possible mechanisms which could be involved in the exercise induced platelet activation are the mobilization of newly formed and more active platelets, thrombin production through the intrinsic coagulation pathway, subendothelial collagen exposure, increases in plasma levels of catecholamines, release of ADP from destroyed erythrocytes and thromboplastins from damaged tissue and activated monocytes.[49–51]

According to Hansen *et al.*[50] the enhancement of the fibrinolytic activity, evidenced by shortened whole blood clot lysis time, is caused by the release of tissue plasminogen activator from endothelium and by an unknown cell mediated component acting in concert with tissue plasminogen activator.

It has been hypothesized that an imbalance between the activation of blood platelets, coagulation pathways and increase of fibrinolytic activity could lead to myocardial infarction, cardiac arrest and be the cause of sudden death after acute exercise, especially in subjects with cardiac risk factors, but also in apparently well-trained individuals.[49] On the other hand, it has been suggested that regular exercise training could result in an improvement in haemostatic balance.[51] As pointed out by Bourey and Santoro,[51] data on the effects of regular physical activity on the coagulation and fibrinolytic systems are sparse and discordant. According to these authors, further studies with improved and more standardized techniques are needed to better define the relationship of exercise to haemostatic mechanisms. Based on these data, it appears that more attention should be paid to detect subjects at risk of developing anormal haemostatic changes and related cardiovascular complications.

### Conclusions

From the authors' own studies and literature data, there are thus a number of elements suggesting that strenuous exercise triggers an inflammatory response similar to that accompanying infection or tissue injury. The main indices of this inflammatory reaction include an acute phase response, leucocyte mobilization and activation, release of inflammatory mediators, complement activation, free radical production, cellular infiltrates and haemostatic changes. It has been hypothesized that the initiating events of IRE could be the release of cellular debris from damaged muscles and/or adjacent connective tissue, and the activation of the contact system due to shear stress to endothelium. These factors could act in concert with endotoxaemia in extremely long-term exercise such as triathlon competition. Although these hypotheses appear plausible, the precise mechanisms and sequence of events of IRE remain to be elucidated. Because in most cases, IRE has no pathological consequences, it has been suggested that the main purpose of IRE is to promote tissue repair. However, severe and persistent exercise induced inflammatory response is thought to be detrimental. Further studies are required to detect, on the basis of objective criteria, the individuals for whom IRE can be considered as a potential hazard. In this view, the influence of several factors such as age, physical training, environmental conditions, nutrition and anti-inflammatory drugs on IRE should be investigated further.

### References

1. Haralambie G. Serum glycoproteins and physical exercise. *Clin Chim Acta* 1969; **26**: 287–291.
2. Haralambie G, Flaherty DK. Serum glycoprotein levels in athletes in training. *Experientia* 1970; **26**: 959–960.
3. Liesen H, Dufaux B, Hollmann W. Modifications of serum glycoproteins the days following prolonged physical exercise and the influence of physical training. *Eur J Appl Physiol* 1977; **37**: 243–254.
4. Cannon JG, Kluger MJ. Endogenous pyrogen activity in human plasma after exercise. *Science* 1983; **220**: 617–619.
5. Taylor C, Rogers G, Goodman C, *et al.* Hematologic, iron-related, and acute-phase protein response to sustained strenuous exercise. *J Appl Physiol* 1987; **62**: 464–469.
6. Cannon JG, Orencole SF, Fielding RA *et al.* Acute phase response in exercise: interaction of age and vitamin E on neutrophils and muscle enzyme release. *Am J Physiol* 1990; **259**: R1214–R1219.
7. Cannon JG, Meydani SN, Fielding RA *et al.* Acute phase response in exercise. II. Association between vitamin E, cytokines, and muscle proteolysis. *Am J Physiol* 1991; **260**: R1235–R1240.
8. Weight LM, Alexander D, Jacobs P. Strenuous exercise: analogous to the acute-phase response? *Clin Sci* 1991; **81**: 677–683.
9. Singh A, Smoak BL, Patterson KY, LeMay LG, Veillon C, Deuster PA. Biochemical indices of selected trace mineral in men: effect of stress. *Am J Clin Nutr* 1991; **53**: 126–131.
10. Kushner I. The phenomenon of the acute phase response. *Ann NY Acad Sci* 1982; **389**: 39–48.
11. Dinarello CA. Interleukin-1 and the pathogenesis of the acute phase response. *N Engl J Med* 1984; **311**: 1413–1418.
12. Cannon JG, Fielding RA, Fiatarone MA, Orencole SF, Dinarello CA, Evans WJ. Increased interleukin-1b in human skeletal muscle after exercise. *Am J Physiol* 1989; **257**: R451–455.
13. Herbaczynska-Cedro K, Staszewska-Barczak J, Janczewska H. Muscular work and the release of prostaglandin-like substances. *Cardiovasc Res* 1976; **10**: 413–420.
14. Nowak J, Vennmalm A. Effect of exercise on human arterial and regional venous plasma concentrations of prostaglandin E. *Prostaglandins Med* 1978; **1**: 489–497.
15. Demers LM, Harrison TS, Halbert DR, Santen RJ. Effect of prolonged exercise on plasma prostaglandin levels. *Prostaglandins Med* 1981; **6**: 413–418.
16. Symons JD, Theodossy SJ, Longhurst JC, Stebbins CL. Intramuscular accumulation of prostaglandins during static contraction of the cat triceps surae. *J Appl Physiol* 1991; **71**: 1837–1842.
17. Rotto DM, Massey KD, Burton KP, Kaufman MP. Static contraction increases arachidonic acid levels in gastrocnemius muscles of cats. *J Appl Physiol* 1989; **68**: 2721–2724.
18. Stebbins CL, Carretero OA, Mindroiu T, Longhurst JC. Bradykinin release from contracting skeletal muscle of the cat. *J Appl Physiol* 1990; **69**: 1225–1230.
19. Dufaux B, Order U. Complement activation after prolonged exercise. *Clin Chim Acta* 1989; **179**: 45–50.
20. Dufaux B, Order U. Plasma elastase-α1-antitrypsin, neopterin, tumor necrosis factor, and soluble interleukin-2 receptor after prolonged exercise. *Int J Sports Med* 1989; **10**: 434–438.
21. Northoff H, Berg A. Immunologic mediators as parameters of the reaction to strenuous exercise. *Int J Sports Med* 1991; **12**: S9–S15.
22. Dufaux B, Order U, Liesen H. Effect of short maximal physical exercise on coagulation, fibrinolysis, and complement system. *Int J Sports Med* 1991; **12**: S38–S42.
23. Camus G, Pincemail J, Duchateau J *et al.* Complement and polymorphonuclear neutrophil activation during submaximal exercise in man. *Arch Int Physiol Biochem* 1991; **100**: P14.
24. Smith JK, Chi DS, Krish G, Reynolds S, Cambron G. Effect of exercise on complement activity. *Ann Allergy* 1990; **65**: 304–310.
25. Viti A, Muscettola M, Paulescu L, Bocci V, Almi A. Effect of exercise on plasma interferon levels. *J Appl Physiol* 1985; **59**: 426–428.
26. Sprenger H, Jacobs C, Nain M *et al.* Enhanced release of cytokines, interleukin-2 receptors, and neopterin after long-distance running. *Clin Immunol Immunopathol* 1992; **63**: 188–195.
27. Schaefer RM, Kokot K, Heidland A, Plass R. Jogger's leukocytes. *N Engl J Med* 1987; **316**: 223–224.
28. Kokot K, Schaefer RM, Teschner M, Gilge U, Plass R, Heidland A. Activation of leukocytes during prolonged physical exercise. *Adv Exp Med Biol* 1988; **240**: 57–63.
29. Pincemail J, Camus G, Roesgen A *et al.* Exercise induces pentane production and neutrophil activation in humans. Effects of propranolol. *Eur J Appl Physiol* 1990; **61**: 319–322.
30. Hansen JB, Wilsgard L, Osterud B. Biphasic changes in leukocytes induced by strenuous exercise. *Eur J Apppl Physiol* 1991; **62**: 157–161.
31. Camus G, Pincemail J, Lamy M. Is there an analogy between sepsis and strenuous physical exercise in the process of neutrophil activation in man: a working hypothesis. In: Vincent JL, ed. *Update in Intensive Care and Emergency Medicine.* Berlin, Heidelberg: Springer Verlag, 1991; 206–212.
32. Camus G, Pincemail J, Ledent M *et al.* Plasma levels of polymorphonuclear elastase and myeloperoxidase after uphill walking and downhill running at similar energy cost. *Int J Sports Med* 1992; **13**: 443–446.
33. Hikida RS, Staron RS, Hagerman FC, Sherman WN, Costill DL. Muscle fiber necrosis associated with human marathon runners. *J Neurol Sci* 1983; **59**: 185–203.
34. Jones DA, Newham DJ, Round JM, Tolfree SEJ. Experimental human muscle damage: morphological changes in relation to other indices of damage. *J Physiol* 1986; **375**: 435–448.
35. Round JM, Jones DA, Cambridge G. Cellular infiltrates in human skeletal muscle: exercise induced damage as a model for inflammatory muscle disease? *J Neurol Sci* 1987; **82**: 1–11.
36. Smith LL. Acute inflammation: the underlying mechanism in delayed onset muscle soreness? *Med Sci Sports Exer* 1991; **23**: 542–551.
37. Camus G, Sondag D, Maggipinto J, *et al.* Mobilisation des leucocytes lors de l'exercice dynamique sous-maximum. *Arch Int Physiol Biochim* 1991; **99**: 419–423.
38. McCarthy DA, Dale MM. The leucocytosis of exercise. A review and model. *Sports Med* 1988; **6**: 333–363.
39. Davies KJA, Quintanilha AT, Brooks GA, Packer L. Free radicals and tissue damage produced by exercise. *Biochem Biophys Res Commun* 1982; **107**: 1198–1205.
40. Dillard CJ, Litov RE, Savin WM, Dumelin EE, Tappel AL. Effects of exercise, vitamin E and ozone on pulmonary function and lipid peroxidation. *J Appl Physiol* 1978; **45**: 927–932.
41. Jackson MJ, Edwards RHT, Symons MCR. Electron spin resonance studies of intact mammalian skeletal muscle. *Biochim Biophys Acta* 1985; **847**: 185–190.
42. Sjödin B, Westing YH, Apple FS. Biochemical mechanisms for oxygen free radical formation during exercise. *Sports Med* 1990; **10**: 236–254.
43. Duthie GG, Robertson JD, Maughan RJ, Morrice PC. Blood antioxidant status and erythrocyte lipid peroxidation following distance running. *Arch Biochem Biophys* 1990; **282**: 78–83.
44. Gohil K, Viguie C, Stanley WC, Brooks GA, Packer L. Blood glutathione oxidation during human exercise. *J Appl Physiol* 1988; **64**: 115–119.
45. Maughan RJ, Donnelly AE, Gleeson M, Whiting PH, Walker KA, Clough PJ. Delayed-onset muscle damage and lipid peroxidation in man following a downhill run. *Muscle & Nerve* 1989; **12**: 332–336.
46. Sumida S, Tanaka K, Kitao H, Nakadomo F. Exercise-induced lipid peroxidation and leakage of enzymes before and after vitamin E supplementation. *Int J Biochem* 1989; **8**: 835–838.
47. Kanter MM, Nolte LA, Hollozy JO. Effects of an antioxidant vitamin mixture on lipid peroxidation at rest and postexercise. *J Appl Physiol* 1993; **74**: 965–969.
48. Wheeler ME, Davis GL, Gillespie WJ, Bern MM. Physiological changes in hemostasis associated with acute exercise. *J Appl Physiol* 1986; **60**: 986–990.
49. Röcker L, Drygas WK, Heyduck B. Blood platelet activation and increase in thrombin activity following a marathon race. *Eur J Appl Physiol* 1986; **55**: 374–380.
50. Hansen JB, Wilsgard L, Olsen JO, Osterud B. Formation and persistence of procoagulant and fibrinolytic activities in circulation after strenuous physical exercise. *Thromb Haemost* 1990; **64**: 385–389.
51. Bourey RE, Santoro SA. Interactions of exercise, coagulation, platelets, and fibrinolysis—a brief review. *Med Sci Sports Exer* 1988; **20**: 439–446.
52. Thomsen BS, Rodgaard A, Tvede N *et al.* Levels of complement receptor type one (CR1, CD35) on erythocytes, circulating immune complexes and complement C3 splits products C3d and C3c are not changed by short-term physical exercise or training. *Int J Sports Med* 1992; **13**: 172–175.
53. Smith JA, Telford RD, Baker MS, Hapel AJ, Weidemann MJ. Cytokine immunoreactivity in plasma does not change after moderate endurance exercise. *J Appl Physiol* 1992; **73**: 1396–1401.
54. Bosenberg AT, Brock-Utne JG, Gaffin SL, Wells MT, Blake GTW. Strenuous exercise causes systemic endotoxemia. *J Appl Physiol* 1988; **65**: 106–108.
55. Brock-Utne JG, Gaffin SL, Wells MT *et al.* Endotoxaemia in exhausted runners after a long-distance race. *S Afr Med J* 1988; **73**: 533–536.
56. Hasson SM, Daniels JC, Divine JG *et al.* Effect of ibuprofen on muscle soreness, damage and performance: a preliminary investigation. *Med Sci Sports Exer* 1993; **25**: 9–17.
57. Camus G, Pincemail J, Deby-Dupont G, Deby C, Juchmès-Ferir A, Lamy M. Effects of methylprednisolone on exercise-induced increase of plasma level of polymorphonuclear elastase and myeloperoxidase in man. Preliminary results. *Mediators of Inflammation* 1993; **2**: 323–326.
58. Kuipers A, Keizer HA, Verstappen FTJ, Costill DL. Influence of a prostaglandin-inhibiting drug on muscle soreness after eccentric work. *Int J Sports Med* 1985; **6**: 336–339.
59. Kihlström M, Salminen A, Vihko V. Prednisolone decreases exercise-induced acid hydrolase response in mouse skeletal muscle. *Eur J Appl Physiol* 1984; **53**: 53–56.
60. Salminen A, Kihlström M. Protective effect of indomethacin against exercise-induced injuries in mouse skeletal muscle fibers. *Int J Sports Med* 1987; **8**: 46–49.
61. Hellsten Y, Ahlborg G, Jensen-Urstad M, Sjödin B. Indication of *in vivo* xanthine oxidase activity in human skeletal muscle during exercise. *Acta Physiol Scand* 1988; **134**: 159–160.
62. Hellsten Westing Y, Ekblom B, Sjödin B. The metabolic relation between hypoxanthine and uric acid in man following maximal short-distance running. *Acta Physiol Scand* 1989; **137**: 341–345.
63. Brunori M, Falcioni G, Fioretti E, Giardina B, Rotilio G. Formation of superoxide in the autoxidation of the isolated $\alpha$ and $\beta$ chains of human hemoglobin and its involvement in hemichrome precipitation. *Eur J Biochem* 1975; **53**: 99–104.
64. Babior BM. The respiratory burst of phagocytes. *J Clin Invest* 1984; **73**: 594–601.
65. Fantone JC, Ward PA. Role of oxygen-derived free radicals and metabolites in leucocyte-dependent inflammatory reactions. *Am J Pathol* 1982; **107**: 397–418.
66. Deby C. La biochimie de l'oxygène. *La Recherche* 1991; **22**: 56–64.
67. Deby C. Metabolism of polyunsaturated fatty acids, precursors of eicosanoids. In: Curtis-Prior PB, ed. *Prostaglandins: Biology and Chemistry of Prostaglandins and Related Eicosanoids.* Edinburgh: Churchill Livingstone, 1988; 11–36.
68. Warren JA, Jenkins RR, Packer L, Witt EH, Armstrong RB. Elevated muscle vitamin E does not attenuate eccentric exercise-induced muscle injury. *J Appl Physiol* 1992; **72**: 2168–2175.
69. Amelink GJ, van der Wal WAA, Wokke JHJ, van Asbeck BS, Bär PR. Exercise-induced muscle damage in the rat: the effect of vitamin E deficiency. *Pflügers Arch* 1991; **419**: 304–309.
70. Frei B, Stocker R, Ames BN. Antioxidant defenses and lipid peroxidation in human blood plasma. *Proc Natl Acad Sci* 1988; **85**: 9748–9752.
71. Camus G, Felekidis A, Pincemail J *et al.* Blood levels of reduced/oxidized glutathione and plasma concentration of ascorbic acid during eccentric and concentric exercises of similar energy cost. *Arch Int Physiol Biochim Biop* (in press).

**Received 27 July 1993; accepted 29 July 1993**
